# Coping Mechanisms and Resilience in Later Life: The Role of Family and Friendship Ties in Diverse Settings

**DOI:** 10.1093/geroni/igaa057.1928

**Published:** 2020-12-16

**Authors:** Feinian Chen, Rashmi Gupta, Zhenmei Zhang

## Abstract

The papers in this symposium explore different aspects of social ties and how they act as critical coping mechanisms in the face of negative circumstances in later life. Using data from diverse settings, including China, Singapore, and the U.S., these papers underscore the importance of strong family and friendship ties, as they offer older adults with strong protection against social isolation and adverse health outcomes. Gupta and Pilai explore the similarity and differences in coping strategies/resilience among a diverse group of 30 U.S. older adults. Results point to the saliency of support from friends, regardless of race/ethnicity. Visaria addresses the relationship between the expression of loneliness and objective measures of social networks among older adults in Singapore. The findings shed light on how meaningful companionship and desired social connection offer powerful buffers against isolation in later life. Ruan and Chen explore which types of social ties offer the strongest protection when Chinese older adults are coping with the aftermath of negative life events. Findings point to the need to look beyond filial obligations and to consider the interplay among various forms of social support, including family, friends and the broader community. Zhang et al. examine the role of family and friendship ties in a rural Chinese community where many older adults were left behind by migrant children. The results suggest that those who are isolated from friends experience more depressive symptoms while those with close-knit friendship ties are the most resilient.

